# Clinical Usefulness of Autoantibodies in Idiopathic Inflammatory Myositis

**DOI:** 10.3389/fimmu.2015.00331

**Published:** 2015-06-25

**Authors:** Ignacio García-De La Torre

**Affiliations:** ^1^Department of Immunology and Rheumatology, Hospital General de Occidente de la Secretaría de Salud, University of Guadalajara, Zapopan, México

**Keywords:** autoantibodies, inflammatory myositis, clinical utility of autoantibodies, myositis-specific antibodies, myositis-associated antibodies

## Introduction

One of the most important characteristics of systemic autoimmune diseases, including idiopathic inflammatory myopathies (IIM), is the immune response to self-antigens manifested by the production of autoantibodies that recognize a variety of cytoplasmic and nuclear antigens. In the last 40 years, autoantibodies of patients with IIM have been investigated; however, some of the fundamental questions related to these antibodies remain unresolved (1).

Indirect immunofluorescence (IIF), in which HEp-2 cells are used as a substrate, is considered the gold standard for detecting antinuclear antibodies (ANA’s) in autoimmune rheumatic diseases. Using this method, specific antibodies that occur exclusively in these diseases were present in a great proportion of patients with myositis (50–80%) (2). It is important to note that patients with myositis more frequently have high titers of these antibodies than patients with overlap syndromes, and low titers usually are present in patients with myopathies associated with neoplasia. In contrast, these autoantibodies are usually absent in patients with inclusion body myositis, dystrophies, and other non-autoimmune myopathies.

In general, even though the diagnostic value of these autoantibodies has been limited, they are useful as diagnostic markers since they closely correlate with certain clinical manifestations of disease and help identify clinical subgroups and to know about the prognosis of the IIM.

Idiopathic inflammatory myopathies has two major types of autoantibody groups: (1) those found primarily in patients suffering from polymyositis (PM) or dermatomyositis (DM) are known as myositis-specific autoantibodies (MSAs); these are not observed in other diseases of connective tissue and are almost completely absent in patients with muscular dystrophies and (2) those that appear in myositis overlap syndromes and in other connective tissue diseases, which correlate with certain clinical and/or pathophysiological conditions of myositis, are known as myositis-associated autoantibodies (MAAs) (3).

## Myositis-Specific Autoantibodies

In general, MSAs are useful markers for clinical diagnosis, classification, and for predicting the prognosis of the IIM. MSAs have two major autoantibody subsets. Probably, the largest and most interesting subset is the anti-cytoplasmic antibodies, which consist of two main groups: those antibodies that are directed to various aminoacyl-tRNA synthetases (ARSs), cytoplasmic enzymes responsible for catalyzing the esterification of a specific amino acid to its cognate tRNA, forming an aminoacyl-tRNA. To each of the 20 amino acids corresponds a unique tRNA.

Autoantibodies against histidyl-tRNA-synthetase (anti-Jo-1) (4) are the most common MSAs that belong to anti-cytoplasmic antibodies and were first described in 1980.These antibodies are found in approximately 25–30% of myositis patients, and they attach histidine to the appropriate tRNA. The anti-Jo-1 antibodies (4) were subsequently found in a group of patients with anti-synthetase syndrome (5), a unique clinical syndrome that includes myositis, interstitial lung disease (ILD), non-erosive arthritis, fever, and characteristic hyperkeratotic lesions along the radial and palmar aspects of the fingers known as “mechanic’s hands.”

Other antibodies from the same subgroup of anti-cytoplasmic antibodies targeting a number of additional ARSs have since been identified, including those recognizing threonyl-tRNA-synthetase (anti-PL-7), alanyl-tRNA synthetase (anti-PL-12), glycyl-tRNA synthetase (anti-EJ), isoleucyl-tRNA synthetase (anti-OJ), asparaginyl-tRNA synthetase (anti-KS), tyrosyl-tRNA synthetase (anti-YRS), and, most recently, phenylalanyl synthetase (anti-Zo) (4). This group of autoantibodies to ARS occurs in approximately 1–5% of patients with myositis (5). It is worth noting that antibodies to one synthetase do not appear to cross-react with other synthetases, but other autoantibodies may occur with anti-synthetase, such as anti-SS-A and anti-SS-B.

The other subsets of anti-cytoplasmic antibodies are the anti-signal-recognition particle antibodies (anti-SRP). This is a non-synthetase antibody, and is found in approximately 4% or less of patients with myositis. SRP is a six-polypeptide complex (72, 68, 54, 19, 14, and 9-kDa) and one 7SL RNA molecule. The cytosolic ribonucleoprotein binds to the endoplasmic reticulum (ER), signaling sequences of elongating polypeptide chains during the synthesis and translocating them to the ER membrane. This autoantibody was first described in 1986 in a “typical PM” patient (6).

Later work showed that autoantibodies could be directed to any of the six polypeptides, as well as to 7SL RNA. The clinical features in the majority of patients with anti-SRP antibodies are unusually severe muscle weakness, dysphagia, and high creatinine kinase levels; poor response to steroids; and a worse prognosis compared to that in patients with other myositis. Furthermore, a large amount of necrotic and regenerating fibers were found in muscle biopsies taken from these patients, who also showed a lower frequency of lymphocytic inflammation than patients affected by other myositis (7).

The other group of MSAs is ANA, which occurs more frequently (approximately 60%) than anti-cytoplasmic antibodies, and it shows a homogeneous, speckled, or nucleolar pattern. Anti-Mi-2 is the only ANA that is also considered a MSA and was first described in 1976 (8). In 1995, their cognate antigens were cloned and autoantibodies to Mi-2 immunoprecipitated a nuclear complex consisting of up to eight subunits. Currently there are two known protein targets, Mi-2α (240-kDa) and Mi-2β (218-kDa), of the Mi-2 antigen.

Both forms of Mi-2 are different molecules that contain a set of helicase motifs with a similar function as nuclear helicases. Mi-2β and histone deacetylases form a protein complex called nucleosome-remodeling deacetylase complex, (9) which may be involved in gene transcription through histone acetylation, causing a remodeling of the nucleosome structure. In most studies, an antibody to Mi-2 has been associated with DM in adult and juvenile patients. It is rarely present in PM and leads to fewer complications related to ILD. In general, patients who have Mi-2 have a good prognosis and show good response to therapy.

A number of other MSAs have been described in different populations of patients with myositis. Clinical usefulness of these autoantibodies has been identified in patients with amyopathic DM and malignant-associated myositis.

In 2005, a specific autoantibody was identified in patients with clinically amyopathic DM (C-ADM), which is a term suggested for patients with typical DM rashes for at least 2 years, but without muscle symptoms. In the early 1990s, these patients were considered to have DM sine myositis. This specific antibody was detected in eight of 15 patients (53%) with C-ADM by immunoprecipitation and immunoblotting. This antibody was termed anti-CADM-140, since it precipitated a 140-kDa protein.

Typical DM rashes were found in the eight patients who had anti-CADM-140 in this study; however, they showed no muscle weakness or muscular pain and had normal serum creatinine kinase levels. In another study of 15 patients who had C-ADM, 13 of them developed ILD; of these, five developed an acute progressive type of ILD. Four of these patients had anti-CADM-140. Therefore, antibody to CADM-140 may serve as a specific serologic marker for patients with myositis and acute ILD (10).

In subsequent studies, the target antigen of CADM-140 was identified using HeLa cell-derived complimentary DNA (cDNA) library. Specifically, the antibody reacted with the “melanoma differentiation-associated gene 5” (*MDA-5*) which encodes an RNA helicase that plays important roles in the innate immune system during RNA virus infection. The protein was expressed in cells transfected with full-length MDA-5 cDNA, confirming the identity of *MDA-5* as the CADM-140 autoantigen.

In addition, an ELISA using recombinant *MDA-5* protein as the antigen showed an analytical sensitivity of 85% and analytical specificity of 100% compared with the results of the “gold standard” immunoprecipitation assay. The antigen was also useful for identifying patients with C-ADM and/or rapidly progressive ILD (11). By using indirect IIF, this antibody shows granular or reticular cytoplasmic staining resembling cytoskeletal components.

Targoff et al. described a previously unknown autoantibody that recognizes a 155-kDa protein (p155) in patients with DM. This antibody is present mainly in patients with adult and juvenile DM and, interestingly, in patients with cancer-associated DM. Since it was present only in one patient with systemic lupus erythematosus (SLE) among a large group of non-myositis controls, this is considered a DM-specific antibody (12).

In another study, Targoff determined that the transcriptional intermediary factor 1-γ (TIF1-γ) is the target antigen of anti-p155 antibody. Interestingly, intracytoplasmic systems of protein translation are the primary immunological targets of PM-specific autoantibodies, while the targets of DM-specific autoantibodies are nuclear transcription factors. This suggests that PM and DM have different pathogenic mechanisms or different production systems of MSAs.

A Japanese group studying 52 myositis patients found an autoantibody that immunoprecipitated 155- and 140-kDa proteins in seven of them (13%). These patients (anti-p155/p140-positive) showed more frequent typical DM rashes than the other patients, but none had ILD. This autoantibody seems to be the same as the autoantibody to p155 reported by Targoff et al. However, the Japanese group claims they are not the same, since the 155-kDa band was always associated with a 140-kDa band in their study.

A preliminary study of United States patients with juvenile DM described a new anti-MJ antibody that targets a 140-kDa protein. It was identified as nuclear matrix protein NXP-2 (13). Moreover, Gunawardena et al., studying UK patients with juvenile DM, found an autoantibody to a 140-kDa protein that was identical to the anti-MJ (anti-NXP-2) antibody. They screened 162 juvenile myositis patients and found 23% of them positive for anti-NXP-2 antibody. The autoantibody was detected only in patients with juvenile DM. It has been confirmed that this antibody is different from anti-CADM-140 and anti-p155.

Betteridge et al. (14) reported a new autoantibody against small ubiquitin-like modifier activating enzyme (SAE) in patients with DM. The post-translational modification of specific proteins such as protein kinases and transcription factors is greatly influenced by small ubiquitin-like modifiers. The authors studied 266 patients with myositis, 250 with other rheumatic diseases, and 50 healthy controls. Of these, 11 (4%) had anti-SAE, which immunoprecipitated both 40 and 90 kDa proteins; all of these patients suffered from DM and showed classical skin lesions (13).

A new autoantibody associated with necrotizing myopathy was also described by Christopher-Stine et al. in a preliminary report. The authors studied immunoprecipitations from ^35^ *S*-methionine-labeled HeLa cell lysates of 225 patients with myositis and reported necrosis on muscle biopsy in 38 patients but no perifascicular atrophy or red-rimmed vacuoles; 26 patients showed unidentified MSAs (15).

In 16 of these 26 patients, an anti-200/100 antibody, which immunoprecipitated both 200-and 100-kDa proteins, was found. In subsequent studies, the 100-kDa autoantigen was identified as the 3-hydroxy-3-methylglutaryl-coenzyme A reductase (HMGCR), the pharmacologic target of statin medications. Additional experiments indicated that the 200-kDa autoantigen is the dimeric form of HMGCR (15).

The majority of these patients (63%) had a history of exposure to statins prior to developing muscle weakness. It is not clear if exposure to this drug intensifies this myopathy, but these results may provide useful information on the immune-mediated mechanisms of necrotizing myopathy.

A list of these MSAs and MAAs is outlined in Table 1.

**Table 1:** **Autoantibodies in myositis, their target antigens, M.W., frequency, and clinical significance**.

Antibodies	Nature of target antigens	MW of antigens in kDa	Frequency In IIM (%)	Clinical significance
**MYOSITIS-SPECIFIC AUTOANTIBODIES (MSAs)**				
anti-ARS
anti-Jo-1	Histidyl-tRNA synthetase	50/52	15–20	Antisynthetase syndrome (myositis, interstitial lung disease, polyarthritis, mechanic’s hand Raynaud’s phenomenon, fever)
anti-PL-7	Threonyl-tRNA synthetase	83	5–10	
anti-PL-12	Alanyl-tRNA synthetase	70	<5	
anti-EJ	Glycyl-tRNA synthetase	75	5–10	
anti-OJ	Isoleucyl-tRNA synthetase	145	<5	
anti-KS	Asparaginyl-tRNA synthetase	63	<5	
anti-Zo	Phenylalanyl-tRNA synthetase	52	<1	
anti-YRS	Tyrosyl-tRNA synthetase	59	<1	
anti-SRP	Signal recognition particle	9–72	<4	Necrotizing myopathy
anti-Mi-2	Mi-2α/Mi-2β helicase family proteins	240/218	5–10	Dermatomyositis
anti-CADM-140	Melanoma differentiation-associated gene 5 (MDA-5)	140	50 (C-ADM)	Specific in C-ADM and acute ILD
Anti-p155(/p140)	Transcriptional intermediary factor 1γ (TIF1-γ)	155/140	20 (DM)	Dermatomyositis, especially in cancer-associated dermatomyositis
anti-MJ	Nuclear matrix protein (NXP-2)	140	<23	Juvenile dermatomyositis
anti-PMS1	DNA repair mismatch enzyme	120	7.5	DM and PM
anti-SAE	Small ubiquitin-like modifier activating enzyme	40/90	<4	Dermatomyositis
anti-HMGCR	HMG-CoA reductase	200/100	62%	Statin-induced autoimmune-necrotizing myopathy
**MYOSITIS-ASSOCIATED AUTOANTIBODIES (MAAs)**				
anti-U1RNP	U1 small nuclear RNP	11–70	10	Overlap myositis, MCTD
anti-SS-A/Ro	Ro-52/TRIM21 and Ro-60 proteins	52/60	≥35%	ILD in IIM patients
anti-Ku	DNA-PK regulatory subunit	70/80	20–30	Polymyositis–SSc overlap in Japanese
anti-PM-Scl	Nucleolar protein complex of 11–16 proteins	75/100	10–15	Polymyositis–SSc overlap

*ARS, aminoacyl-tRNA synthetases; C-ADM, clinically amyopathic dermatomyositis; ILD, interstitial lung disease; HMGCR, 3-hydroxy-3 methylglutaryl-coenzyme A reductase; DNA-PK, DNA-dependent protein kinase; MCTD, mixed connective tissue disease; SSc-scleroderma. MW, molecular weight; kDa, kilodalton*.

## Myositis-Associated Autoantibodies

The more common MAAs includes anti-U1-RNP, anti-SS-A/Ro, anti-PM/Scl, and anti-Ku, which are found in patients with myositis and various overlap syndromes (16).

U1-RNP is a small nuclear ribonucleoprotein consisting of U1-snRNA and nine proteins: 70 K (70-kDa), A (34-kDa), B’/B (29/28-kDa), C (22-kDa), D (16-kDa), E (13-kDa), F (12-kDa), and G (11-kDa). It is part of a spliceosome that eliminates intervening sequences from pre-mRNA to produce mature mRNA. Anti-U1-RNP antibody is a serological marker for mixed connective tissue disease, very common in definite scleroderma (SSc), SLE, PM/DM, and overlapping syndromes.

Anti-SS-A/Ro antibody is found in some autoimmune diseases such as Sjögren’s syndrome, SLE, SSc, and IIM. Anti-SS-A/Ro recognizes two distinct antigens, with molecular weights of 52-kD (Ro-52/TRIM21) and 60-kDa (Ro-60), respectively. Each antibody has a different clinical association. Anti-Ro-52/TRIM21 is significantly more prevalent in SSc and myositis than anti-Ro-60, while isolated anti-Ro-52/TRIM21 may be found in up to 37% of patients with myositis, often in correlation with anti-Jo-1 reactivity. The presence of anti-SS-A/Ro-52/TRIM21 antibodies is associated with more severe ILD, particularly in patients with anti-synthetase syndrome (13).

Anti-PM/Scl antibodies are a heterogeneous group of autoantibodies directed to several proteins of the nucleolar PM/Scl macromolecular complex. PM/Scl-75 and PM/Scl-100 are the two main autoantigenic protein components and are named based on their apparent molecular weights. These antibodies are associated with PM-SSc overlap syndrome in approximately 15% of the patients, and may be present also in other autoimmune diseases (16).

The antibody to Ku was reported for the first time in Japanese patients suffering from PM-SSc overlap syndrome, and it was considered a serologic marker of it. However, studies carried out in other countries have yielded different results. In the United States, anti-Ku antibody was found mostly in SLE patients. The difference in the results may be due to the use of different assay systems and the differences in ethnic or genetic background of the patients studied (16). The target antigen of anti-Ku antibody is a heterodimer of 70- and 80-kDa proteins that is thought to be an activation subunit of DNA-dependent protein kinase. The Ku antigen has key roles in various cellular processes, including DNA double-strand break repair.

## Conclusion

In the last 20 years, several MSAs and MAAs, and their target autoantigens, clinical phenotypes, and histological features have been identified. This group of autoantibodies may yield useful insights into their diagnosis, prognosis, and treatments.

Testing for the majority of these autoantibody specificities is already commercially available through different immunoassays. These methods aid to establish the correct diagnosis in cases in which an autoimmune muscle disease is suspected.

## Conflict of Interest Statement

The author declares that this manuscript and associated research was done in the absence of any commercial or financial relationships that could be construed as a potential conflict of interest.
